# Rain and Sun Create Slippery Layers in Eastern Pacific Fresh Pool

**DOI:** 10.5670/oceanog.2019.217

**Published:** 2019-06-14

**Authors:** Andrey Y. Shcherbina, Eric A. D’Asaro, Ramsey R. Harcourt

**Affiliations:** Applied Physics Laboratory, University of Washington, Seattle, WA, USA.

## Abstract

An autonomous Lagrangian float equipped with a high-resolution acoustic Doppler current profiler observed the evolution of upper-ocean stratification and velocity in the Eastern Pacific Fresh Pool for over 100 days in August–November 2016. Although convective mixing homogenized the water column to 40 m depth almost every night, the combination of diurnal warming on clear days and rainfall on cloudy days routinely produced strong stratification in the upper 10 m. Whether due to thermal or freshwater effects, the initial strong stratification was mixed downward and incorporated in the bulk of the mixed layer within a few hours. Stratification cycling was associated with pronounced variability of ocean surface boundary layer turbulence and vertical shear of wind-driven (Ekman) currents. Decoupled from the bulk of the mixed layer by strong stratification, warm and fresh near-surface waters were rapidly accelerated by wind, producing the well-known “slippery layer” effect, and leading to a strong downwind near-surface distortion of the Ekman profile. A case study illustrates the ability of the new generation of Lagrangian floats to measure rapidly evolving temperature, salinity, and velocity, including turbulent and internal wave components. Quantitative interpretation of the results remains a challenge, which can be addressed with high-resolution numerical modeling, given sufficiently accurate air-sea fluxes.

## INTRODUCTION

The ocean’s response to wind forcing in the presence of rotation has been the subject of study for over 100 years, since the work of Ekman (1905), but the problem still appears to be far from solved. Nuances arising from time-dependent coupling of upper-ocean shear, turbulence, waves, and stratification are plentiful, and not fully accounted for in some of the widely used idealizations, such as slab-layer or quasi-steady approximations (Polton et al., 2005; Wenegrat and McPhaden, 2016). One of the most illustrative examples of such coupling is the so-called “slippery layer” phenomenon, created when the strong surface buoyancy input due to diurnal heating or precipitation traps the wind momentum and causes rapid acceleration of the near surface currents, even in moderate wind conditions (Kudryavtsev and Soloviev, 1990; Anderson et al., 1996). Recent studies have also revealed links between the variations of wind-driven turbulence modulated by diel heating and the dynamics of upper-ocean submesoscale motions (Dauhajre and McWilliams, 2018).

**FIGURE 1.**
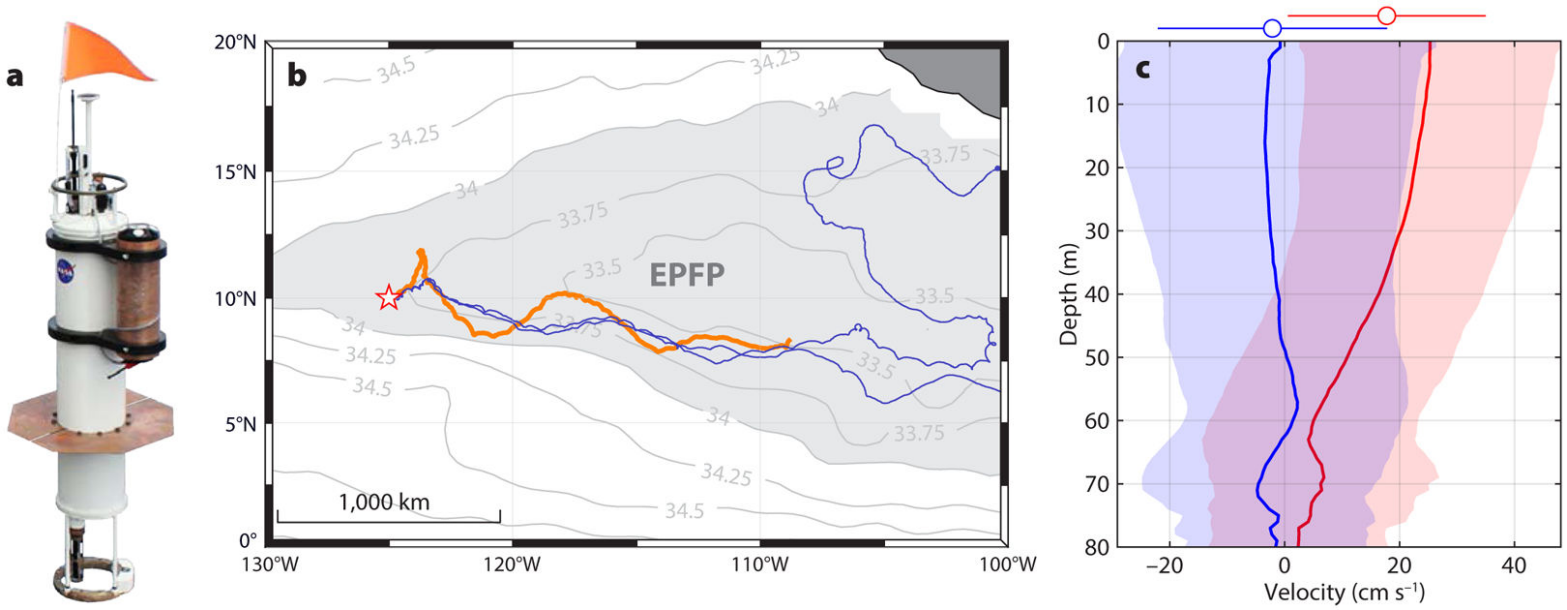
(a) SPURS-2 Lagrangian float design. (b) Lagrangian float drift progress (orange track), August 26–December 12, 2016. The background shows the mean annual surface salinity based on Aquarius satellite data, with the 34.0 isohaline roughly outlining the extent of the Eastern Pacific Fresh Pool (EPFP). The location of the SPURS-2 central mooring (Farrar and Plueddemann, 2019, in this issue) is marked with a star. Tracks of two NOAA-AOML drifters over the same time period are shown in blue. (c) Eastward (red) and northward (blue) upper-ocean advection observed by the float. Means and one standard deviation intervals are shown by solid lines and light shading, respectively. The means and standard deviation intervals of the float’s net progression velocities are shown with circles and bars above the axes. Mixed layer Lagrangian float (MLF) trajectories followed an average of the horizontal advection in the top 60 m of the water column.

Wind-driven dynamics are known to be important in shaping the Eastern Pacific Fresh Pool (EPFP; Alory et al., 2012), a relatively fresh area in the tropical Pacific (Figure 1b). Although the origins of the EPFP can be traced to a band of heavy precipitation beneath the Intertropical Convergence Zone (ITCZ), wind-driven (Ekman) advection has been shown to control the location and extent of the EPFP low-salinity patch (Yu, 2014). These basin-scale conclusions have been drawn from the bulk mixed-layer approximation of Ekman dynamics.

However, upper layers of the EPFP may not be well mixed all of the time. Frequent rainfall creates highly stratified shallow “lenses” that last several hours before they are incorporated into the bulk of the mixed layer (Drushka et al., 2019, in this issue). Although rain beneath the ITCZ is frequent, it is not continuous. On clear days, robust thermal stratification is created via solar heating, only to be mixed away during the period of night-time cooling and ensuing convective mixing. Therefore, the EPFP undergoes typical tropical diurnal warm layer cycling (e.g., Moulin et al., 2018), but with an added complication of pronounced rainfall effects. Resulting variability of upper-ocean stratification can be expected to have a profound effect on surface boundary layer turbulence and, by extension, on Ekman layer development.

One focus of the second NASA Salinity Processes in the Upper-ocean Regional Study (SPURS-2) experiment was the investigation of the role of the small-scale, transient, wind- and rain-driven dynamics in the evolution of the near-surface structure of the EPFP. Here, we present the first look at the observations and modeling of these small-scale dynamics.

## LAGRANGIAN FLOAT OBSERVATIONS

The SPURS-2 experiment took place in 2016–2017 in the EPFP. Nearly 200 elements of moored, self-navigating, and drifting instrumentation formed a distributed autonomous observing system around the nominal “central” mooring site at 10°N, 125°W. Lindstrom et al. (2017) give a detailed account of the overall experiment design and the interconnected roles played by the individual parts of the observing system.

A mixed layer Lagrangian float (MLF; Figure 1a; D’Asaro et al., 2003) served as a focal point of the freely drifting (Lagrangian) instrument array during the later part of the rainy season (August 26–December 12, 2016). Accurate and highly adaptable automatic buoyancy control, flexible mission planning, and relatively heavy payload distinguish MLFs from other float designs (e.g., APEX/Argo floats also used in SPURS; see Lindstrom et al., 2017). For SPURS-2, the MLF was equipped with high-accuracy dual Sea-Bird CTD sensors and an upward-looking Nortek Signature1000 acoustic Doppler current profiler (ADCP) to observe the evolution of the upper-ocean velocity structure.

The ability to measure velocity is a unique and new feature of the SPURS-2 Lagrangian float. For this purpose, the MLF was equipped with a 1 MHz Signature1000 broadband ADCP with four beams in a standard symmetric Janus configuration pointing upward 25° off the ADCP axis, and a fifth axial (vertical) beam. The ADCP was set up to interleave the long-range, low-resolution (LR) and high-resolution (HR) pulse-coherent sampling on the four slanted beams; the vertical beam was LR only. Detailed description of LR and HR sampling modes and data processing techniques can be found in Shcherbina et al. (2018). LR sampling was configured with 30 one-meter cells and the nominal single-ping radial velocity noise standard deviation of 9 cm s^−1^. The actual usable range of LR measurements was typically 20–25 m, depending on the varying amount of scatterers in the water. LR sampling over the course of the float’s dive can be used to infer the mean vertical profile of horizontal velocity in a manner similar to the lowered or glider-based ADCP (Visbeck, 2002; Todd et al., 2011). HR sampling was much finer in resolution, with 256 three-centimeter range cells, giving a maximum range of 8.18 m and corresponding to 6 mm s^−1^ ambiguity velocity. The noise variance of single-ping HR radial velocity measurements varied greatly with the varying signal correlation, but was typically on the order of 2–4 mm s^-1^. With its improved accuracy and resolution, HR sampling enables new ways of visualizing fine-scale turbulent velocities (see Box 1).

The main objective of the float’s mission was to collect detailed observations of stratification and shear across the upper-ocean boundary layer within EPFP. It was thus programmed to cycle continuously through the upper ocean at ~2 cm s^−1^ vertical profiling speed. The profiles typically reached 60 m depth, slightly deeper than the 50 m maximum (March) climatological mixed layer thickness, according to Monthly Isopycnal & Mixed-layer Ocean Climatology (MIMOC; Schmidtko et al., 2013). Occasional deeper profiles (to 80–120 m) were also included. The temperature and salinity profiles extended all the way to the surface with the use of an additional surface temperature-salinity (STS) probe at the top of the float (Murphy et al., 2008) operating above 25 m depth, thereby resolving the shallow surface layers described here. These high-resolution profiles were enabled by 1 Hz STS sampling, and a profiling speed that was much slower than typical Argo float profiles (~10 cm s^−1^), achieved through active control of the MLF profiling speed using precise ballasting control. During SPURS-2, the slow profiling efforts were partially frustrated by large (up to 300 g) buoyancy changes due to the nocturnal interference of fish (Lien et al., 2008), which sometimes created gaps in otherwise regular profiling.

The float’s horizontal movement can be seen as approximating the mean advection of the upper 60 m layer of ocean overlying the main pycnocline. Onboard LR ADCP observations (Figure 1c) showed that this layer contained the bulk of the eastward upper-ocean advection in the EPFP along the float track. At the same time, observations showed the advection to be sheared in the vertical (rather than slab-like) and variable in time (Figure 1c). The vertical shear was likely associated with recurrent stratification of the upper ocean produced by diurnal heating and frequent precipitation (see the next section). Presence of this shear needs to be taken into account when calculating regional balances of upper-ocean freshwater fluxes that maintain the EPFP. It is also important to keep it in mind when interpreting drifter observations in the area. For example, eastward advection of the NOAA-Atlantic Oceanographic and Meteorological Laboratory (AOML) Surface Velocity Program (SVP) drifters drogued at 15 m and deployed alongside the float (Volkov et al., 2019, this issue) was ~35% faster than that of the MLF, reflecting the mean upper-ocean shear (Figure 1c).

To provide meteorological context for the MLF observations, the float was accompanied by a Liquid Robotics SV-2 Wave Glider (Daniel et al., 2011) that typically stayed within an 11 ± 6 km radius of the float for the duration of its SPURS-2 drift (Lindstrom et al., 2017). The Wave Glider carried an Airmar PB200 Weather Station on a 1 m mast to measure the basic surface meteorology variables (wind speed and direction, air temperature, and pressure). Remaining surface forcing parameters were obtained from remote sensing of precipitation (30 min, 0.1° Integrated Multi-satellitE Retrievals for GPM [IMERG] V05 product; Huffman, 2017) and hourly atmospheric reanalysis heat fluxes (ERA5, European Centre for Medium-Range Weather Forecasts, 2017). Remote-sensing and reanalysis parameters were spatially interpolated to the MLF position.

## RECURRING NEAR-SURFACE STRATIFICATION AND SHEAR

**FIGURE 2.**
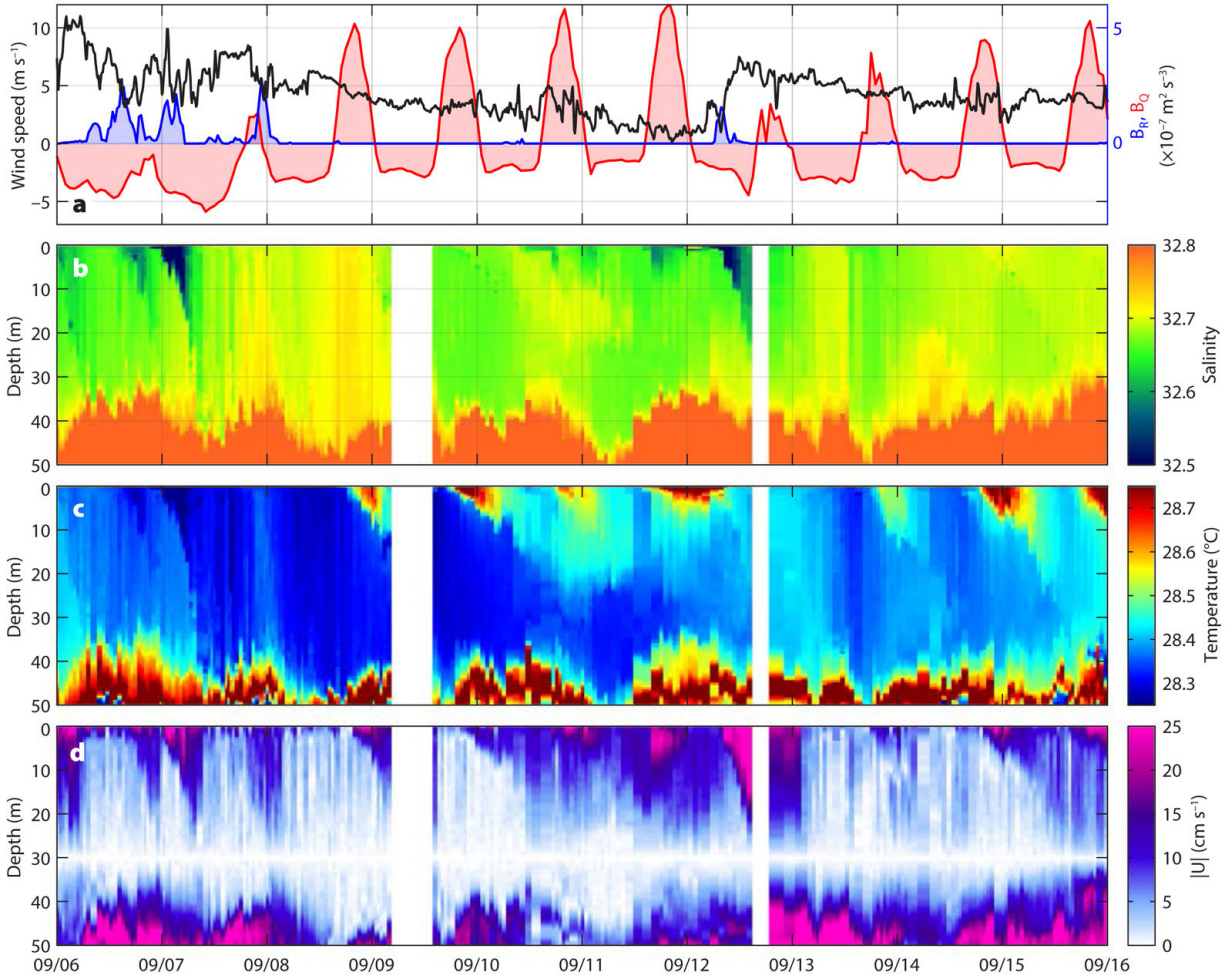
A 10-day sample of upper-ocean stratification and shear observed with the SPURS-2 MLF. (a) Surface forcing: wind speed (black, Wave Glider observations), and surface buoyancy fluxes due to precipitation (blue, IMERG) and due to the net heat flux into the ocean (red, from ERA5); for reference, buoyancy flux of 5 × 10^−7^ m^2^ s^−3^ corresponds roughly to 7.8 mm hr^−1^ precipitation or 650 W m^−2^ net heat flux. Lagrangian float observations of upper-ocean (b) salinity, (c) temperature, and (d) magnitude of horizontal current relative to 30 m. Individual MLF profiles are plotted in (b)-(d). UTC dates for 2016 are shown.

Figure 2 shows a 10-day sample of the float measurements along with the observed wind speed and the estimated surface buoyancy fluxes due to rainfall and heating. During sunny days (September 8–11 and September 14–16), the upper 10 m warmed during the afternoon, with the warm layer thickening and cooling during the following night (Figure 2c). This is the familiar diel cycle (Price et al., 1986), with a diurnal warm layer and nocturnal convection. During two rain events (September 6 and 12), a similar near-surface stratification appeared (Figure 2b), but was due to reduced salinity. This is an example of a freshwater lens, a common occurrence in the EPFP (Drushka et al., 2019, this issue).

In the entire 107-day record, 68 days showed at least one stratification event with maximum density anomaly relative to the mixed layer interior mode exceeding 0.05 kg m^−3^. Of these, 47 days (69%) showed stratification due to diurnal heating, and 44 days (65%) due to rain. During 23 days (34%), periods of both thermal and freshwater stratification were present. Typical surface velocity anomalies relative to 30 m during these near-surface stratification events were 20–30 cm s^−1^ (Figure 2d). In many cases, surface salinity stratification was observed in the absence of measurable precipitation in the IMERG record (e.g., September 10–11 in Figure 2b), and the other way around (e.g., September 8). We do not aim to conduct a detailed comparison of IMERG precipitation with the upper-ocean freshwater content changes here, as such a study needs to be conducted in a more systematic way. The discrepancy, however, highlights the limitations of this precipitation product at small spatial and temporal scales and the need for further investigation of the scales and patterns of rain variability over the ocean (e.g., Thompson et al., 2016).

**FIGURE 3.**
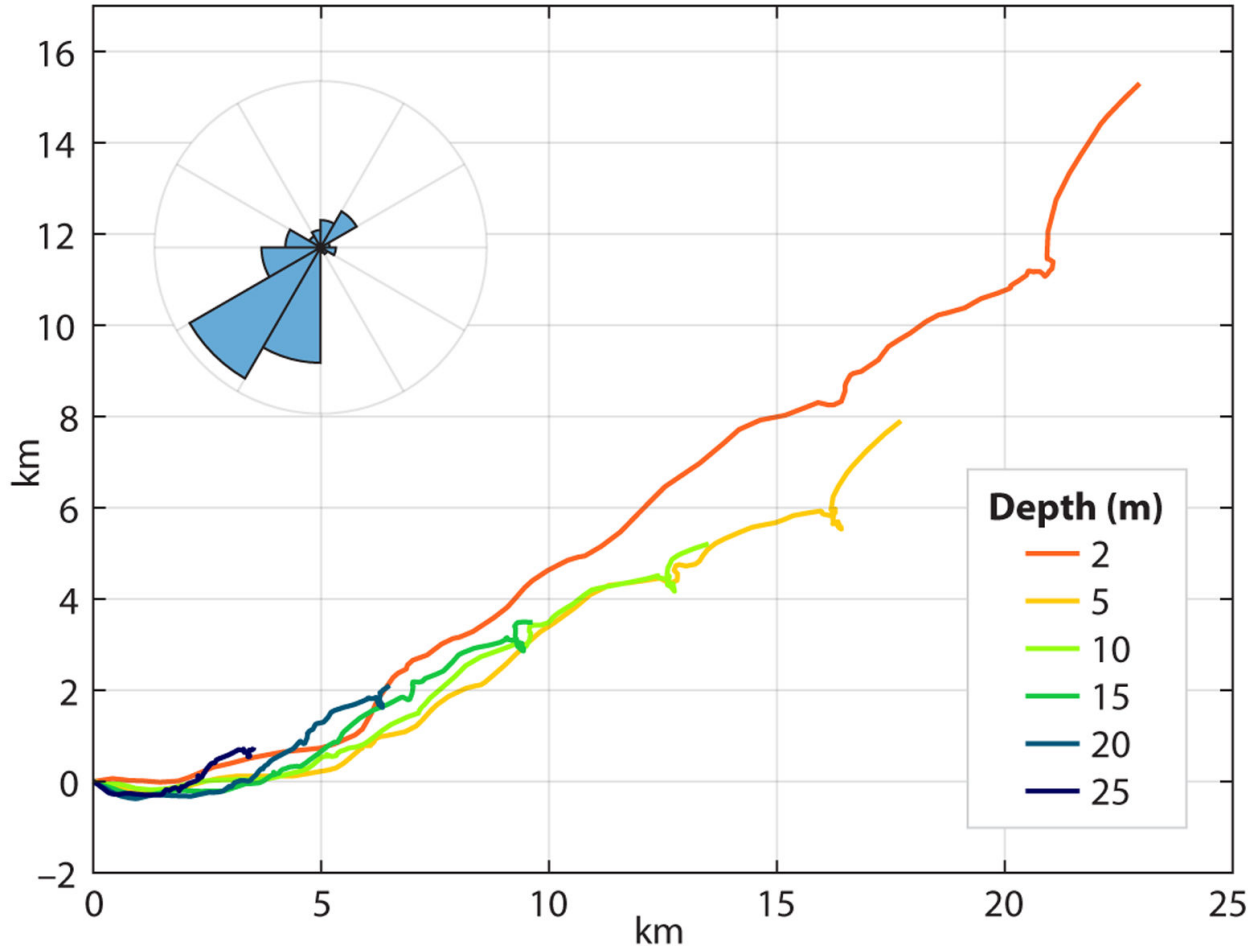
Progressive vector diagram showing the integral advection of the upper-ocean layers relative to 30 m depth over a 10-day period (September 6–16, 2016, same as shown in Figure 2). The inset shows the histogram of wind direction over the same period based on Wave Glider observations. Meteorological wind direction is shown; predominant wind was from southwest.

Regardless of whether temperature or salinity caused the near-surface stratification, the shallow buoyant layers quickly accelerated downwind at speeds reaching 25–30 cm s^−1^ relative to the interior of the mixed layer (Figure 2c). Such rapid acceleration under modest wind speed (5 m s^−1^) is the reason for the term “slippery layer” (Kudryavtsev and Soloviev, 1990). These strong near-surface currents last only a few hours, which is much shorter than the inertial period (~71 hour) at this latitude, so they do not rotate significantly during their lifetimes. Over the 10-day period shown, the average current due to these multiple slippery layers was about 3 cm s^−1^ (1% of the vector-mean wind speed of 3 m s^−1^) or about 25 km relative to 30 m depth over 10 days (Figure 3). Because the average Ekman transport must be perpendicular to the wind stress, these slippery layers are expected to strongly distort the average current profile in a downwind direction in the upper 10 m and thus crosswind and upwind in the deeper layers.

## OCEAN RESPONSE TO A RAIN EVENT

**FIGURE 4.**
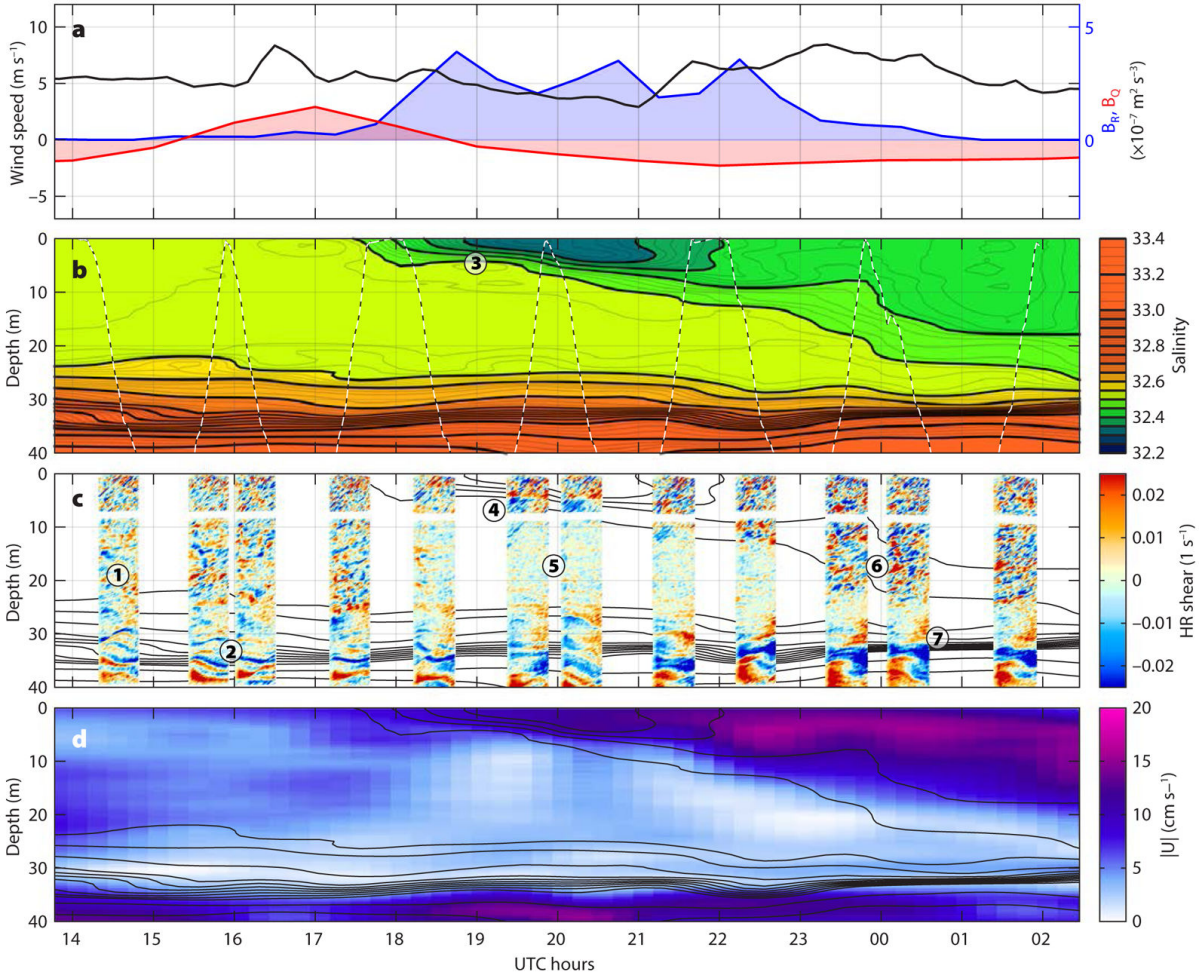
Detail of the upper-ocean response to rainfall October 8–9, 2016. (a) Surface forcing: wind speed (black, Wave Glider observations) and surface buoyancy fluxes due to precipitation (blue, IMERG) and due to the net heat flux into the ocean (red, from ERA5). Lagrangian float observations of upper-ocean (b) salinity, (c) fine-scale shear (see Box 1 for details on shear visualization), and (d) magnitude of horizontal current relative to 30 m. The same salinity contours are shown in black in (b)–(d) for reference. Circled numbers mark different turbulence and stratification regimes in the upper-ocean boundary layer, as discussed in the text. Compare with the large-eddy simulation in Figure 5. Local noon is about 20:30 UTC.

A particularly clear and illustrative example of ocean response to one rain event was captured by an MLF on October 8–9, 2016 (Figure 4). Throughout the event, the wind (Figure 4a) was nearly steady at 5 ± 2 m s^−1^ from the east, later switching to the southeast. Rain (Figure 4a) started at about 18:00 UTC with 23 mm of rainfall occurring over the next eight hours (according to IMERG). In response, a ~4 m thick rain lens formed with a salinity (Figure 4b) about 0.2 g kg^−1^ fresher than that of the mixed layer interior. This fresh layer immediately began to thicken even as the rain continued, so that by the end of the rainfall (02:00 UTC), the layer was ~20 m thick, nearly reaching the bottom of the original mixed layer. This thickening diluted the freshwater lens by entraining saltier water from the underlying mixed layer so that the salinity anomaly continuously decreased, despite the rain, to only 0.05 g kg^−1^ by the end of the event. Freshwater input of the eight-hour rain event estimated from the upper-ocean salinity profiles observed with the MLF was 43 mm of freshwater equivalent. For comparison, integral IMERG rainfall accumulation was only 23 mm (53%) for the same period. This discrepancy is not unexpected, as the IMERG product represents a broad-scale average of precipitation that does not resolve the variability of convective rain events in the ITCZ (Thompson et al., 2019, this issue).

As the freshwater layer was formed, it accelerated downwind, and its relative velocity (Figure 4d) increased to about 10 cm s^−1^ in a few hours. The velocity anomaly subsequently spread downward, following the thickening of the fresh layer. The momentum input from the wind continued, so the velocity anomaly of the fresh layer maintained about the same magnitude despite the layer thickening. The sheared interface underlying the growing fresh layer progressively increased in thickness as well.

The HR velocity observations (Figure 4c) provide more detailed information on the small-scale velocity structure associated with the formation and mixing of a rain lens. We interpret these observations as follows. Prior to the rainfall (14:00–18:00 UTC), HR velocity observations show clear distinctions between the rapidly fluctuating turbulent shear in the mixed layer above 20 m (marked (1) in Figure 4c), and the more time-coherent and intense internal wave shear in the pycnocline below (2). As the rainfall creates a strongly stratified low-salinity layer (3) at the surface, the strong stratification confines the surface wind forcing to this layer. Within this layer, the turbulent shear continues (4), but beneath it the turbulence decays rapidly (compare (1) to (5)). The strong shear maintains the turbulence at the bottom of the fresh layer (6), allowing the entrainment and mixing that deepens the rain lens. Throughout this time, the internal wave shear at the base of the mixed layer (7) continues with little change.

Decay of turbulence under temperature- and salinity-stratified capping layers is a known phenomenon, observed, among others, by Brainerd and Gregg (1993), Smyth et al. (1997), Sutherland et al. (2016), and Moulin et al. (2018). Despite some differences between the heat- and rain-induced capping cases (the latter tend to be more abrupt), these previous observations produced similar e-folding timescales of turbulence decay on the order of tens of minutes. Even though these timescales were of the same order of magnitude as the local buoyancy periods, no clear correlation between the two could be established (Smyth et al., 1997). In all observations, the turbulence decay was concluded to be only partially free, suggesting that the surface capping does not remove all the sources of turbulence in the deeper layers. In particular, it seems likely that shear, such as that observed here, could be an additional source. Observations of upper-ocean boundary layer turbulence decay may therefore be a particularly valuable resource for elucidating the indirect sources and sinks of turbulence and improving their representation in boundary-layer mixing models. Our SPURS-2 observations provide a new visual insight into the process of turbulence evolution. Observed decay rate of turbulence at 15–20 m corresponds to e-folding scale of ~30 minutes, which is about twice the buoyancy period in the mixed layer (70 min at buoyancy frequency *N* ~1.5 × 10^−3^ s^−1^), and also similar to the previously reported values.

**FIGURE 5.**
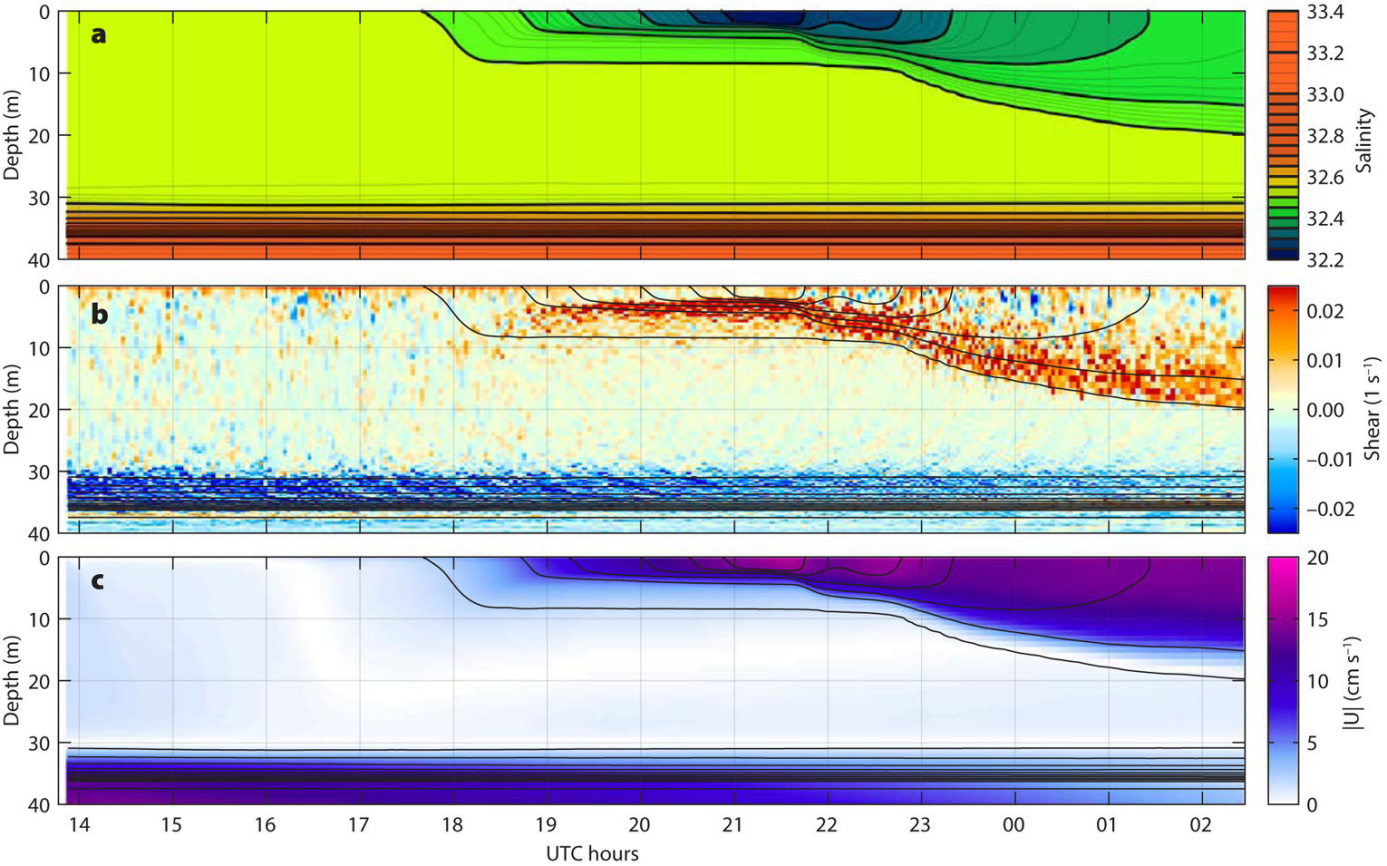
Large-eddy simulation of the upper-ocean response to a rainstorm event on October 8–9, 2016. The MLF observations of the same event are shown in Figure 4. (a) Domain-averaged salinity. (b) Vertical shear at an arbitrary horizontal grid location. (c) Magnitude of horizontal current relative to 30 m. The same salinity contours are shown in black in (b)–(d) for reference. All axes and color maps match their respective counterparts in Figure 4b-d.

Lagrangian float observations, such as those shown in Figure 4, show characteristic signatures of the surface boundary layer dynamics involved in the ocean’s response to surface forcing, but cannot describe them fully. To aid interpretation of the observations, we carry out high-resolution numerical simulations of the same rain event (Figure 5) and use these simulations to explore the relevant physics. Unlike the simulations reported by Clayson et al. (2019, in this issue), the forcing is not well known in our case, due to the lack of in situ measurements of air-sea fluxes. Instead, we rely on a combination of nearby in situ Wave Glider wind observations, remote sensing of precipitation, and meteorological reanalysis to derive a plausible forcing scenario (see Box 2). While fully realistic and illustrative, these simulations are not meant for quantitative model-data comparison due to uncertainty of the forcing.

Direct numerical simulations of upper-ocean boundary layer turbulence would need to account for the very large range in spatial scales, from about 100 m, or several times the boundary layer thickness, to a few millimeters or less, the smallest Kolmogorov dissipation scale, and extend for many integral timescales of the surface boundary layer flow to study the response to such unsteady forcing. This cannot be presently done directly with even the largest computers. Instead, a large-eddy simulation (LES) approach is taken, where only the larger-scale physics are explicitly simulated and the smaller scales are modeled using turbulence closure methods. Box 2 provides details of our LES configuration.

The overall evolution of salinity and velocity structure during the October 8–9, 2016, rain event (Figure 4) is reproduced by the simulation (Figure 5), but with notable deviations in detail. The initial fresh layer forms and mixes downward with similar geometries as well as salinity and velocity anomalies to those observed. Small-scale model shear variations (Figure 5b) are initially spread across the mixed layer, but become confined to the fresh layer, in a manner similar to that seen in the MLF observations. Below the fresh layer, the shear decreases, as in the observations. Simulated downward mixing of the freshwater lens is generally slower than that observed, particularly during the temporary decrease of wind speed observed by the Wave Glider between 19:00 and 21:30 UTC. Simulated mixing increases after 22:00 UTC, but the downward spreading of the freshwater lens continues to lag the observations. Discrepancy is likely due to the inaccurate wind forcing in the model: during the event, the Wave Glider was 30 km away from the MLF, substantially further than the nominal 10 km radius. Therefore, it may not have captured the increase of wind speed associated with the rain event. In fact, Wave Glider sensors did not observe any surface freshening during this period, suggesting that it may have missed the rain event entirely.

The LES may also provide insight into new potentially relevant dynamics. Patterns of upward-propagating shear layers in the marginally stratified sheared surface boundary layer immediately preceding formation of the fresh surface layer, and throughout the deepening process (Figure 5b, after about 20:00 UTC), are remarkably similar to structures studied in nocturnal atmospheric boundary layers (Sullivan et al., 2016), where they are associated with temperature fronts and vortical structures responsible for vertical turbulent transport. Observational verification of such structures is still pending.

## DISCUSSION

These preliminary analyses show the roles of alternating heat and freshwater fluxes in forming strong yet intermittent near-surface stratification in the EPFP. Despite the very different dynamics of these mechanisms, their overall impacts on the density structure and its evolution are quite similar. Together, they create stratification in the top 50 m on a large fraction (>60%) of the days, so that typical mixing depths are a few meters to a few tens of meters, clearly shallower than the 25–40 m climatological mixed layer depth. Accordingly, the changes in the upper ocean due to air-sea fluxes, sea surface temperature, and salinity anomalies caused by heat flux and rainfall, as well as the Ekman velocities caused by wind stress, will be larger than those predicted by using climatological mixed layer depth. This difference will enhance oceanic feedback to atmospheric forcing.

The observations described here demonstrate the rapid advances in autonomous ocean observations. The combination of the Lagrangian float, which provided measurements in a reference frame that minimizes advective effects, and the Wave Glider, which provided local meteorological forcing information, demonstrates the potential to collect detailed observations entirely autonomously. The addition of the new generation ADCPs to the float demonstrates the ability to autonomously make detailed velocity observations of both the overall upper-ocean boundary layer velocity structure and the smaller-scale turbulence. We anticipate that additional processing will allow these fine-scale velocity measurements to be quantitatively interpreted (Thomson et al., 2015). Numerical large-eddy simulations provide invaluable dynamical context for interpretation of the observed phenomenology of upper-ocean boundary layer evolution. Direct model-data comparison is inevitably limited at some level of detail in timing and structure by uncertainties in the ocean surface forcing responsible for driving the vertical mixing processes at the float. A major ongoing challenge is therefore to develop and refine observational techniques that allow high-quality autonomous measurements of surface heat fluxes, wind stress, and precipitation in conjunction with underwater sampling of the ocean’s responses. Our SPURS-2 observations show the potential for long-term multi-instrument coordinated studies of air-sea interaction in a wide variety of environments, particularly those that are not otherwise accessible.

## BOX 1. NEW INSTRUMENT–NEW VISUALIZATION

During the MLF ascent or descent through the water, the ADCP measures series of high-resolution (HR) relative velocity profiles, each extending over the 8 m range above the float (for the particular HR setup used during SPURS-2). At a typical float profiling speed of 2–5 cm s^−1^ and 1 Hz ADCP sampling rate, consecutive profiles largely overlap in depth, although over 10–20 minutes, a wider depth range is covered. At the same time, improved single-ping accuracy of HR measurements makes each profile individually meaningful and allows us to resolve time-space variability of small-scale turbulent velocities (Shcherbina et al., 2018). Visualization of the detailed structure of this variability with respect to real depth presents substantial challenges.

A straightforward time-depth color-coded “standard” plot (Figure B1a) is informative but it cannot be extended for more than a few hours. The displayed swath width is set by the sampling parameters (ADCP range and float profiling speed) and cannot be scaled independently. Because of that, the swath becomes too narrow to discern details (either in print or on a screen) as it grows longer (Figure B1a). Alternatively, the profiling ADCP observations can be seen as a series of top-to-bottom velocity profiles measured by each of the ~250 ADCP cells. These profiles can be presented as color-coded “straightened” swaths (Figure B1b,d), which convey largely the same visual information about the velocity field variability in time and depth, but in a more compact form. The straightened swaths can be plotted at each profile’s mean time (as in Figure 4c), and their width can be scaled arbitrarily for best visual presentation regardless of the time extent of the plot. Note that in either case, each observation is plotted at its accurate depth, but the meaning of the horizontal axes is different. In the standard display, observations are plotted at their actual times; in the straightened presentation, they are spaced uniformly around the mean profile time using a scaled range (or ADCP cell number), as illustrated in Figure B1b,d. The latter display still conveys a sense of time variability of the observed fields at a given depth, because different ADCP cells sample a given depth at different times. However, only approximate timing of the observations and intervals between them can be inferred from such a plot. If presentation of the actual timing is important, the standard display may be preferable, as in Shcherbina et al. (2018).

## BOX 2. LARGE EDDY SIMULATION SETUP

Our simulations use the NCAR LES (Sullivan and Patton, 2011). A horizontally periodic domain (216 m × 216 m, with horizontal grid resolution *dx* = *dy* = 1.5 m) extends vertically to a radiating bottom boundary at 72 m. The vertical grid resolution is variable, ranging from the finest (0.2 m) near the surface and at the top of the pycnocline, and increasing to as much as 1.2 m elsewhere. The time step varies during the simulation with numerical stability constraints, but is typically on the order of a second. The model is initialized with the temperature, salinity, and horizontal velocity profiles observed by the MLF at 14:40 UTC on October 8, just prior to the rain event. The model is forced with the surface wind stress derived from the Wave Glider wind observations, IMERG precipitation, and ERA5 heat fluxes. In order to match the actual freshwater anomaly observed during the rain event, IMERG precipitation rates were scaled up by a factor of two. The LES equations include surface gravity wave forcing via the Craik-Leibovich vortex force due the interaction of the shear of the Stokes drift and the wave-averaged Eulerian current (McWilliams et al., 1997; Harcourt and D’Asaro, 2008). The time-varying Stokes drift profile is computed for a fully developed Pierson-Moskowitz spectrum following Li and Garrett (1993) for the hourly surface wind time series and interpolated with the surface fluxes to the LES time step.
